# Agomelatine Ameliorates Cognitive and Behavioral Deficits in Aβ-Induced Alzheimer’s Disease-like Rat Model

**DOI:** 10.3390/medicina61081315

**Published:** 2025-07-22

**Authors:** Raviye Ozen Koca, Z. Isik Solak Gormus, Hatice Solak, Burcu Gultekin, Ayse Ozdemir, Canan Eroglu Gunes, Ercan Kurar, Selim Kutlu

**Affiliations:** 1 Department of Physiology, Faculty of Medicine, Necmettin Erbakan University, 42080 Konya, Turkey; igormus@gmail.com (Z.I.S.G.); ayseozdemir422@gmail.com (A.O.); skutlu@erbakan.edu.tr (S.K.); 2 Department of Physiology, Faculty of Medicine, Kütahya Health Sciences University, 43020 Kütahya, Turkey; hhaticesolak@gmail.com; 3 Department of Histology, Faculty of Medicine, Necmettin Erbakan University, 42080 Konya, Turkey; dilaygltekin@yahoo.com.tr; 4 Department of Medical Biology, Faculty of Medicine, Necmettin Erbakan University, 42080 Konya, Turkey; cananeroglu88@gmail.com (C.E.G.); ekurar@erbakan.edu.tr (E.K.)

**Keywords:** agomelatine, experimental Alzheimer’s disease model, hippocampal neurogenesis, cognitive functions

## Abstract

*Background and Objectives:* Alzheimer’s disease (AD) has become a serious health problem. Agomelatine (Ago) is a neuroprotective antidepressant. This study aimed to assess how Ago influences behavioral outcomes in AD-like rat model. *Materials and Methods:* Forty-eight Wistar albino rats were allocated into four groups: Control (C), Alzheimer’s disease-like model (AD), Alzheimer’s disease-like model treated with Ago (ADAgo), and Ago alone (Ago). Physiological saline was injected intrahippocampally in C and Ago animals, whereas Aβ peptide was delivered similarly in AD and ADAgo rats. On day 15, 0.9% NaCl was administered to the C and AD groups, and Agomelatine (1 mg/kg/day) was infused into ADAgo and Ago rats via osmotic pumps for 30 days. Behavioral functions were evaluated using Open Field (OF), Forced Swim (FST), and Morris Water Maze (MWM) tests. Brain tissues were examined histopathologically. Neuritin, Nestin, DCX, NeuN, BDNF, MASH1, MT1, and MT2 transcripts were quantified by real-time PCR. Statistical analyses were performed in R 4.3.1, with *p* < 0.05 deemed significant. *Results:* In the FST, swimming, climbing, immobility time, and mobility percentage differed significantly among groups (*p* < 0.05). In the MWM, AD rats exhibited impaired learning and memory that was ameliorated by Ago treatment (*p* < 0.05). DCX expression decreased in AD rats but was elevated by Ago (*p* < 0.05). Nestin levels differed significantly between control and AD animals; MT1 expression varied between control and AD cohorts; and MT2 transcript levels were significantly lower in AD, ADAgo, and Ago groups compared to C (all *p* < 0.05). *Conclusions:* Ago exhibits antidepressant-like activity in this experimental AD model and may enhance cognitive function via mechanisms beyond synaptic plasticity and neurogenesis.

## 1. Introduction

Alzheimer’s disease (AD) is a neurodegenerative condition marked by the loss of neurons and synaptic connections, which manifests as declines in cognition and daily functioning. Advanced age is the principal risk factor for such cognitive deterioration across multiple domains, and inherited genetic variants further exacerbate this decline. Therefore, developing interventions that target the underlying contributors to age- and AD-related cognitive loss is essential [1].

At present, approximately 6.9 million individuals aged 65 and older in the United States (U.S.) are affected by AD, a figure that is anticipated to nearly double, reaching an estimated 13.8 million by the year 2060. AD ranks as the fifth leading cause of death among older adults in the U.S. Despite significant scientific efforts, no definitive preventive treatment has been established to date, underscoring the critical need for effective interventions to delay onset and progression of dementia. With over 55 million individuals globally affected by AD and related dementias, developing and implementing strategies to preserve cognitive function has become an urgent public health priority [2].

Unchangeable risk factors for Alzheimer’s include older age, carrying the APOE ε4 gene variant, and a family history of the disease [2]. In addition, up to 40% of cases have been linked to changeable factors such as physical inactivity, smoking, low educational attainment, reduced social and cognitive engagement, hypertension, and poor diet, with midlife obesity, sedentary behavior, and limited schooling being the most prevalent [3].

AD represents a progressive neurodegenerative disorder in which cognitive functions steadily deteriorate, often accompanied by the onset of behavioral and psychiatric disturbances. As the pathology advances, individuals frequently exhibit symptoms ranging from mood disorders such as depression and anxiety to frank psychotic episodes. Recent research indicates that disruptions of neurotransmitter pathways and receptor activity emerge early in AD. Deciphering these underlying biological mechanisms is crucial for developing novel interventions aimed at alleviating the neuropsychiatric manifestations of the disease [4].

A growing body of evidence suggests that depression in later life significantly elevates the risk of developing dementia, particularly AD, with considerable clinical and pathological overlap observed between the two conditions. [5].

Agomelatine (Ago) functions as a melatonin receptor agonist (MT1–MT2) and as a serotonin receptor (5-HT_2_C) receptor antagonist. By engaging melatonin pathways, Ago reestablishes normal circadian rhythms, producing both antidepressant and anxiolytic benefits. Its inhibition of 5-HT_2_C receptors further modulates monoamine release in the prefrontal cortex, bolstering mood regulation. Because emotional balance and sleep–wake cycles are tightly linked to learning and memory, these mechanisms may help mitigate the cognitive decline and memory impairment seen in neurodegenerative conditions such as AD [6].

The principal role of brain MT1 and MT2 is to govern sleep–wake cycles and circadian regulation. Agonists targeting these receptors not only alleviate disturbances in circadian rhythms and sleep but may also offer therapeutic benefits for mood disorders, AD, and autism spectrum conditions [7]. Studies have demonstrated that MT1 immunoreactivity within the suprachiasmatic nucleus (SCN) declines in both early- and late-stage AD patients compared with age-matched controls [8]. A parallel decrease in melatonin receptor expression has likewise been reported in the hippocampus and retina of individuals with AD [9].

Ago, a novel antidepressant, has been shown to improve mood disturbances and sleep impairment in AD. Recent work highlights its neuroprotective properties—specifically antioxidant and anti-apoptotic actions and its ability to counteract age-related inflammation and oxidative stress [10]. Since advancing age is a major AD risk factor, these effects imply that Ago may also attenuate both the underlying drivers and associated comorbidities of AD [11]. Accordingly, this study was designed to examine effect of Ago on behavior and cognitive performance via neurogenesis in an experimental AD model.

## 2. Materials and Methods

All experimental procedures involving animals were approved by the Local Ethics Committee of the Experimental Medicine Application and Research Center at NE University (Approval No: 2020-014).

### 2.1. Experimental Design and Animals

**Figure 1:**
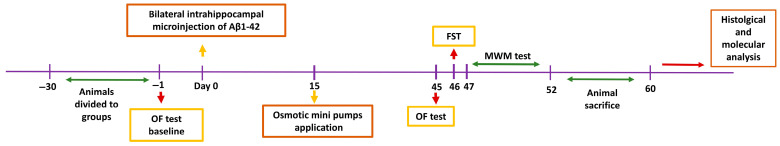
Schematic of the experimental timeline. Rats were allocated to four groups 30 days prior to interventions. Baseline locomotor activity was assessed using the Open Field (OF) test. On day 1, an Alzheimer’s-like state was induced via intrahippocampal Aβ injection. Mini-osmotic pumps were implanted on day 15. The OF test was repeated on day 45, followed by the Forced Swim Test (FST) on day 46. Habituation in the Morris Water Maze (MWM) occurred on day 47, with training trials conducted on days 48–51 and a probe trial on day 52. On day 53, animals were euthanized, and hippocampal tissues were harvested for gene expression and histological evaluation.

A total of 48 male Wistar albino rats were included in this study. The animals were obtained from KONUDAM Experimental Medicine Application and Research Center. The animals were housed under standard laboratory conditions, maintained at a constant room temperature of 22 ± 1 °C, with a 12 h light/dark cycle. They had free access to food and water. The rats were randomly assigned to four groups (*n* = 12 per group): Control (C), Alzheimer’s disease-like model (AD), AD model treated with agomelatine (ADAgo), and agomelatine-only group (Ago) (Figure 1).

### 2.2. Experimental AD Model

The AD model was adapted from previous studies [12,13]. The rats determined to be under anesthesia were placed in a stereotaxic device (Stoelting Stellar Rat Stereotaxic Instrument, Stoelting, Wood Dale, IL, USA) as appropriate. Aβ1-42 peptide (2.2 nmol/10 μL) was injected intrahippocampally into the CA1 region of the hippocampus [14] in the Alzheimer’s model rats (AD and ADAgo). The same amount of physiological saline was injected intrahippocampally into the rats in the C and Ago groups.

### 2.3. Administration of Drugs

The rats were anesthetized, and osmotic pumps were placed subcutaneously 15 days after the amyloid beta injection. In the AG groups, ago dissolved in a dose of 1 mg/kg/day was added to the osmotic pump reservoir for 30 days, and the solvent was added to the other groups in the same manner.

After determining the coordinates with the stereotaxic method in the C and Ago groups, the same amount of physiological serum as Aβ was applied intrahippocampally bilaterally at a concentration of 5 μg/μL. On the 15th day after this application, osmotic mini pumps were placed, and 0.9% NaCl (SF) was administered to the C, and 1 mg/kg/day Ago was applied to the Ago group for 30 days, and then behavioral tests were performed. After determining the coordinates with the stereotaxic method in the AD and ADAgo groups, Aβ was administered intrahippocampally bilaterally at a concentration of 5 μg/μL. On the 15th day after this application, osmotic mini pumps were placed, and 0.9% NaCl (SF) was administered to AD group, and 1 mg/kg/day ago was applied to the ADAgo group for 30 days, and then behavioral tests were performed.

### 2.4. Behavioral and Memory Tests

Following treatment, all animals underwent a battery of behavioral assays: Open Field Test (OF) to assess anxiety and locomotor activity, Forced Swim Test (FST) to gauge depressive-like responses, and Morris Water Maze (MWM) to evaluate learning and memory.

#### 2.4.1. Open Field Test

OF test is used to assess both locomotor activity and anxiety behavior [15]. OF test was administered twice: initially to establish baseline measurements, and again at the conclusion of the study to assess alterations in exploratory behavior and anxiety levels. The apparatus was a square Plexiglas arena (80 × 80 × 30 cm), with a virtual central zone (40 × 40 cm) occupying half the floor area and the remaining peripheral zone. Rats were placed in center and allowed to explore for five minutes while specialized software tracked total distance traveled, average speed, and the number of entries into the central area. Rearing and grooming behaviors were scored by an observer. All testing took place from 09:00 to 12:00 h, and the enclosure was wiped down with ethanol after each session.

#### 2.4.2. Forced Swim Test

This is the most commonly used test in depression research, especially in antidepressant treatment screening. Rats are placed alone in a water-filled cylinder for 15 min (Acclimation phase). After 24 h, they are placed back in the same cylinder for 5 min (Test phase). During the acclimation and test phases, the animals are dried and kept in their cages. In the test phase, the animal’s swimming, climbing, and immobilization times are evaluated [16].

#### 2.4.3. Morris Water Maze Test

The MWM is a standard assay for spatial learning and memory [17]. MWM consists of a pool filled with room temperature water and a platform placed inside. In MWM, the pool is divided into four quadrants. There is a platform in the center of one of these quadrants. Visual cues are used to help animals find the platform in the area where the experiment is conducted. The platform and cues are kept constant in each session to evaluate the cognitive functions of the experimental animal [18,19]. The test was performed for all animals between 9:00 and 12:00 every morning. The general experimental protocol is 6 days. On the first day, a familiarization phase is performed with a visible platform. In each trial, the training phase—which consists of 4 trials per day, starting from a different starting point (quadrant)—is performed for four consecutive days. In the memory test trial on the last day, platform is removed, and the experimental animal is released into the water from a different quadrant. On day 6 (probe trial), the platform was removed, and rats were released from a novel quadrant; swim paths, latency to the former platform site, and time spent in the target quadrant were recorded. All sessions were video-tracked by ceiling-mounted cameras and analyzed using specialized software.

### 2.5. Total RNA Isolation, Reverse Transcriptase (RT) Reaction, and Real-Time Quantitative Polymerase Chain Reaction (qPCR) Analysis

Under light sedation, animals were decapitated, and their hippocampi were rapidly dissected, flash-frozen in liquid nitrogen, and stored at −80 °C for subsequent gene expression analysis. Total RNA was extracted from these samples using TRIzol reagent, and cDNA synthesis was carried out following manufacturer’s instructions. Quantitative PCR was conducted with Sybr Green dye, employing primer pairs for Neuritin, Nestin, Doublecortin (DCX), NeuN, BDNF, MASH1, MT1, MT2, and the reference genes SDHA and RPL13A (see Supplementary Table S1). Melt curve analysis confirmed that each reaction produced a single, specific amplicon (Supplementary Figure S1). All molecular assays adhered to protocols from our previous work [20,21,22,23].

### 2.6. Histological Analysis

All animals were euthanized, and their brains were collected and immediately fixed in 10% formaldehyde. After fixation, tissue blocks were processed through a graded ethanol series for dehydration, cleared in xylene, and embedded in paraffin. Sections of 5–6 µm thickness were cut and mounted on slides, then stained with Hematoxylin and Eosin (H&E), Toluidine-Blue for histological evaluation.

For the hematoxylin and eosin protocol, slides were first incubated at 60 °C for 1 h to enhance tissue adhesion. They were then deparaffinized in xylene for 10 min, followed by rehydration through descending ethanol concentrations (100%, 90%, 80%, and 70%, each for 3 min), with a final rinse in tap water. Sections were immersed in hematoxylin solution for 30 s, rinsed, and then stained in Eosin Y for 15 s before a final wash. After staining, slides were dehydrated, cleared, and coverslipped.

Slides were dehydrated through a graded ethanol series: first 70%, then 80% ethanol for 30 s, 90% ethanol for 1 minute, and 100% ethanol for 2 minutes, followed by a 10 min xylene clearing step. After xylene, sections were air dried under a cover slip. For toluidine blue staining, slides were dipped sequentially in absolute ethanol and 90% ethanol, then placed in toluidine solution for 40 s. Following a rinse in distilled water, coverslips were mounted with Entellan^®^ once dry. Five-micrometer sections from all experimental group were deparaffinized in xylene and rehydrated through descending ethanol concentrations (90%, 80%, 70%, 50%) for fibrosis assessment. Masson’s Trichrome Stain Kit (Lot 072022.036) was applied according to the manufacturer’s instructions. Sections were then rinsed through increasing alcohols and xylene before final mounting in Entellan^®^. For Congo Red staining of amyloid deposits (modified from Ha et al. [24]), deparaffinized slides were stained in Congo Red solution for 15 to 20 min, rinsed in distilled water, and differentiated with five to ten dips in an alkaline ethanol solution (1 mL of 1% NaOH in 100 mL of 50% ethanol). After counterstaining with hematoxylin for 30 s and a one minute tap-water wash, sections were dehydrated in 95–100% ethanol, cleared in xylene, and coverslipped with Entellan^®^.

### 2.7. Statistical Analysis

An a priori power analysis was conducted using G*Power 3.1 software to estimate the minimum sample size required to detect medium effect sizes (f = 0.25) with a significance level of 0.05 and a statistical power of 0.80. The analysis indicated that a minimum of 9 rats per group would be sufficient. However, to increase the robustness and reliability of the findings, particularly given the multiple behavioral and molecular endpoints assessed in this study, 12 rats were included in each group.

Behavioral data were analyzed in R, version 4.3.1 (R Foundation for Statistical Computing, Vienna, Austria). Numerical variables are presented as mean ± standard deviation (SD) when normally distributed and as median (interquartile range) when not. One-way ANOVA was used to match single-timepoint behavioral outcomes such as OF total distance and FST immobility time, as well as for gene expression values. Linear mixed-effects models were used to analyze repeated measures data, particularly the MWM training trials across days, where group and time were modeled as fixed effects, and individual animals as random effects to account for within-subject variability over time. Post hoc pairwise comparisons were performed using Tukey-adjusted least square means when ANOVA results were significant.

Molecular data (gene expression studies) were analyzed using IBM SPSS Statistics for Windows, version 28.0 (IBM Corp., Armonk, NY, USA). Succinate dehydrogenase complex subunit A (SDHA) and ribosomal protein L13A (RPL13A) served as reference genes for normalization. Relative transcript abundance was calculated using the 2^−ΔCt^ method. Group differences were evaluated with one-way ANOVA, and pairwise comparisons were conducted with the Least Significant Difference (LSD) post hoc test. A *p*-value less than 0.05 was regarded as indicating statistical significance in all tests.

## 3. Results

### 3.1. Weight Change

**Figure 2:**
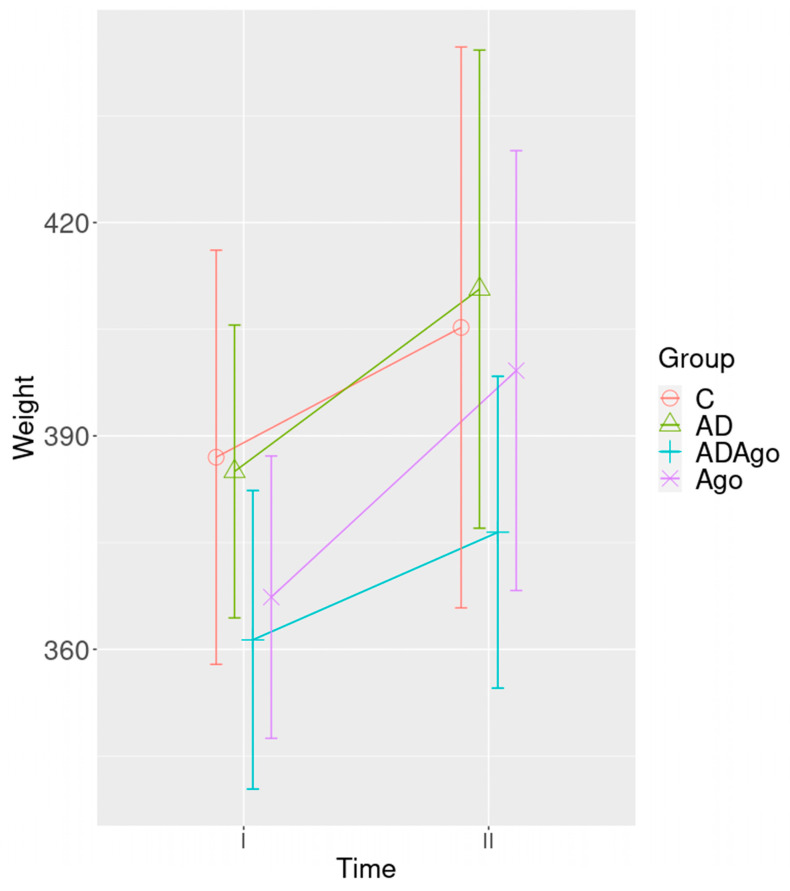
There was no statistically significant difference in body weight changes among the groups when comparing the measurements taken at the start and conclusion of the experiment (*p* = 0.176). C: control; AD: Alzheimer’s disease-like; ADAgo: Alzheimer’s disease–like + agomelatine; Ago: agomelatine.

Analysis of body weight from the start to the end of study revealed no significant main effect of group (*p* = 0.176) nor any group-by-time interaction (*p* = 0.411) (Figure 2).

### 3.2. Open Field Test

**Figure 3:**
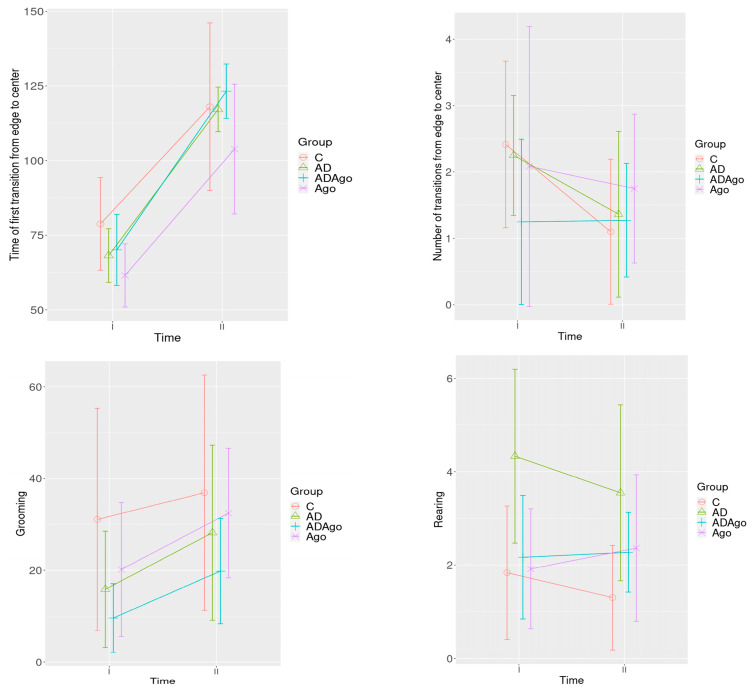
The number transitions from edge to center, the first transition time from edge to center, rearing, and grooming parameters in the OF test. No significant difference was observed for the parameter of number of edge to center transitions in the OF test performed at the beginning and end of the experiment (*p* = 0.098). There was no significant difference between the groups (*p* = 0.125) for the first transition time from the edge to the center, but a significant difference was observed depending on time (*p* < 0.001). Grooming parameter in the OF test performed at the beginning and end of the experiment, no significant difference was observed between the groups (*p* = 0.093) and group–time interaction (*p* = 0.063). Rearing parameter, a significant difference was observed between C and AD (*p* = 0.038), AD and Ago groups (*p* = 0.047) at Time I. C: control; AD: Alzheimer’s disease-like; ADAgo: Alzheimer’s disease-like + agomelatine; Ago: agomelatine.

The number of transitions from the edge to the center, latency to first transition, rearing, and grooming were assessed in the OF. At the initial time point (Time I), rearing differed significantly between C and AD (*p* = 0.038) and between AD and Ago (*p* = 0.047). When comparing baseline and final measurements, grooming showed neither a main effect of treatment (*p* = 0.093) nor a treatment × time interaction (*p* = 0.063). Likewise, the total edge-to-center crossings did not change significantly over study (*p* = 0.098), and latency to first entry remained similar across treatments (*p* = 0.125), although it did decrease significantly over time (*p* < 0.001) (Figure 3).

In the OF, the following parameters were evaluated: number of transitions from edge to the center, time of first transition edge to the center, grooming, and rearing behaviors. (A) The number of edge-to-center transitions showed no statistically significant difference across groups at the beginning and end of the experiment (*p* = 0.098). (B) While no significant group difference was detected for time of first transition edge to the center (*p* = 0.125), a significant effect was observed depending on time (*p* < 0.001). (C) For grooming behavior, there were no statistically significant differences either between the groups (*p* = 0.093) or for the group × time interaction (*p* = 0.063). (D) Regarding rearing behavior at Time I, significant differences were found between the C and AD groups (*p* = 0.038), and also between the AD and Ago groups (*p* = 0.047).

### 3.3. Force Swimming Test

In FST, swimming, climbing, immobilization time, and mobility percentage were analyzed. There is a significant difference between groups in the FST (*p* < 0.001) (Table 1).

**Figure 4:**
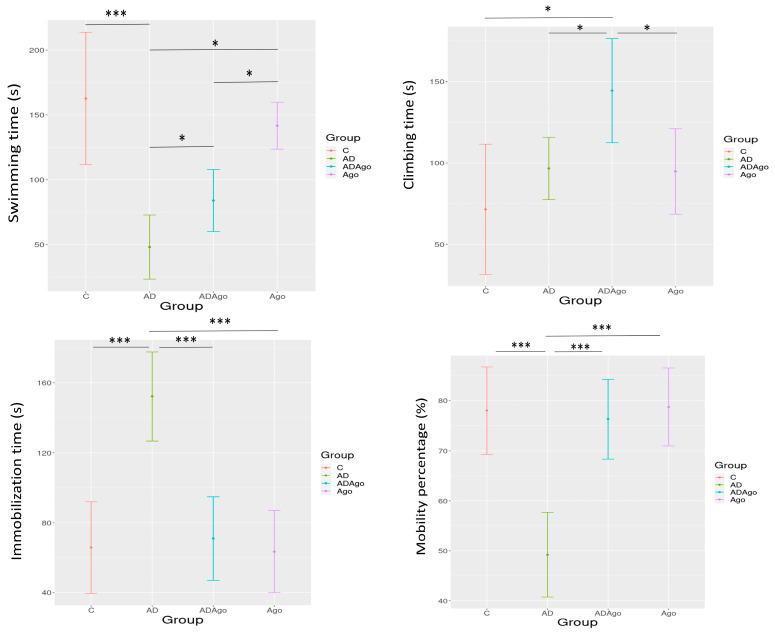
In the FST, swimming, climbing, immobilization time, and mobility percentage were analyzed. A significant difference was observed between C and AD (*p* < 0.001), C and ADAgo (*p* = 0.002), AD and Ago (*p* < 0.001), ADAgo and Ago groups (*p* = 0.023) in the swimming time parameter. There was a significant difference in climbing time parameter between the C and ADAgo (*p* = 0.003), AD and ADAgo (*p* = 0.067), and ADAgo and Ago groups (*p* = 0.047). In FST, the immobilization time was observed to be significant among the groups (*p* < 0.001). There was a significant difference between C and AD, AD and Ago, AD and ADAgo groups in the immobilization time (*p* < 0.001). There was a significant difference among the groups in the percentage of mobility parameter (*p* < 0.001). The percentage of mobility was observed to be significant between C and AD, AD and Ago, AD and ADAgo groups (*p* < 0.001). C: control; AD: Alzheimer’s disease-like; ADAgo: Alzheimer’s disease-like + Agomelatine; Ago: agomelatine, * *p* < 0.05, *** *p* < 0.001.

A significant difference was observed between C and AD (*p* < 0.001), C and ADAgo (*p* = 0.002), AD and Ago (*p* < 0.001), and ADAgo and Ago groups (*p* = 0.023) in the swimming time parameter. There was a significant difference in climbing time parameter between C and ADAgo (*p* = 0.003), AD and ADAgo (*p* = 0.067), and ADAgo and Ago groups (*p* = 0.047). In FST, the immobilization time was observed to be significant among the groups (*p* < 0.001). There was a significant difference between the C and AD, AD and Ago, and AD and ADAgo groups in the immobilization time (*p* < 0.001). There was a significant difference among the groups in the percentage of mobility parameter (*p* < 0.001). The percentage of mobility was observed to be significant between C and AD, AD and Ago, and AD and ADAgo groups (*p* < 0.001) (Figure 4).

### 3.4. Morris Water Maze (MWM) Test

There is a training phase and a test phase in MWM. In the training phase, distance moved and latency to platform were compared in 4 days. In the test phase, distance moved, latency to platform, time spent in platform area, velocity, number of passes through target quadrant, time spent in target quadrant, and first transition time to target quadrant parameters were evaluated.

**Figure 5:**
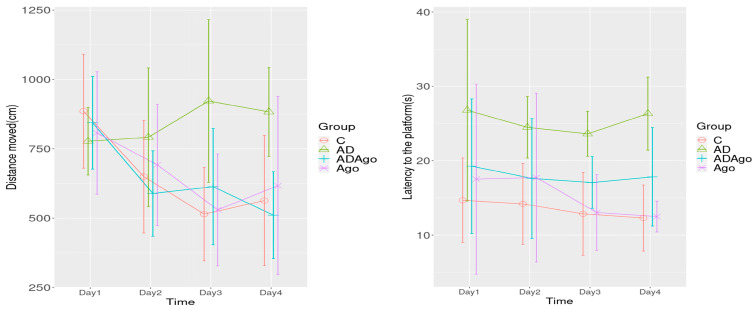
A significant difference was observed among the groups and the group–time interaction in the distance covered parameter in the training phase of MWM (*p* = 0.047). A significant difference was observed between C and AD (*p* = 0.010), AD and Ago groups (*p* = 0.046) on Day 3, and between the AD and ADAgo groups on Day 4 (*p* = 0.017). A significant difference was observed between the groups in the latency to the platform parameter in training phase of MWM (*p* < 0.001). A significant difference was observed between the C and AD groups on Day 1 (*p* = 0.017), Day 2 (*p* = 0.047), Day 3 (*p* = 0.034) and Day 4 (*p* = 0.003), and between AD and Ago groups on Day 3 (*p* = 0.049) and Day 4 (*p* = 0.011) (Figure 5). C: control; AD: Alzheimer’s disease-like; ADAgo: Alzheimer’s disease-like + agomelatine; Ago: agomelatine.

During the training phase a significant difference was observed among the groups and the group–time interaction in the distance covered parameter of MWM (*p* = 0.047). A significant difference was observed between C and AD (*p* = 0.010), AD and Ago groups (*p* = 0.046) on Day 3, and between the AD and ADAgo groups on Day 4 (*p* = 0.017). A significant difference was observed between the groups in the latency to the platform parameter in training phase of MWM (*p* < 0.001). A significant difference was observed between C and AD groups on Day 1 (*p* = 0.017), Day 2 (*p* = 0.047), Day 3 (*p* = 0.034), and Day 4 (*p* = 0.003), and between the AD and Ago groups on Day 3 (*p* = 0.049) and Day 4 (*p* = 0.011) (Figure 5).

There were significant differences among the groups in parameters of distance moved, latency to platform, time spent in target quadrant, and first transition time to target quadrant in the test phase of MWM.

**Figure 6:**
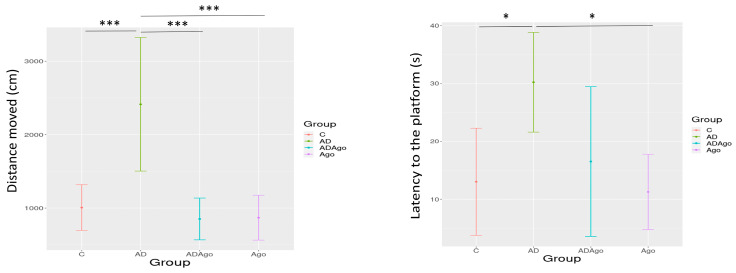

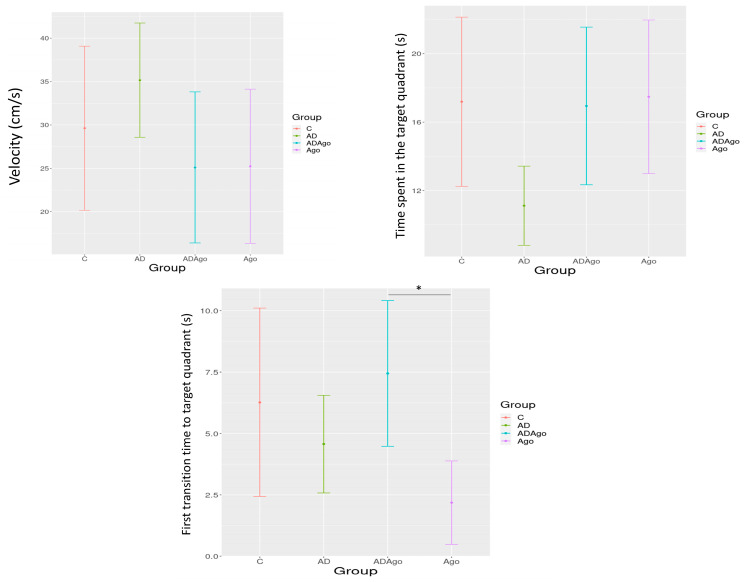
A significant difference between groups C and AD, AD and ADAgo, AD and Ago in the parameter of distance moved during test phase (*p* < 0.001). There was a significant difference between groups C and AD (*p* = 0.011), AD and Ago (*p* = 0.005) in the parameter of latency to platform. There was no significant difference among the groups in the velocity parameter (*p* > 0.05). No significant difference was observed among the groups about the time spent in the target quadrant parameter (*p* > 0.05). Although no statistical difference was observed, when the graph was examined, it was observed that AD group slightly decreased in time spent target quadrant parameter. A significant difference was observed between ADAgo and Ago groups in the parameter of first transition time through target quadrant (*p* = 0.043). C: control; AD: Alzheimer’s disease-like; ADAgo: Alzheimer’s disease-like + agomelatine; Ago: agomelatine. * *p* < 0.05, *** *p* < 0.001.

There was a difference between C and AD, AD and ADAgo, and AD and Ago in distance moved during test phase (*p* < 0.001). There was a significant difference between the groups C and AD (*p* = 0.011) and AD and Ago (*p* = 0.005) in the parameter of latency to the platform during test phase of MWM. There was no significant difference among the groups in velocity parameter during test phase of MWM (*p* > 0.05). No significant difference was observed among the groups about time spent in target quadrant parameter (*p* > 0.05). Although no statistical difference was observed, when the graph was examined, it was observed that AD group slightly decreased in time spent in target quadrant parameter. A significant difference was observed between ADAgo and Ago groups in the parameter of first transition time through target quadrant during test phase of MWM (*p* = 0.043) (Table 2, Figure 6).

### 3.5. Gene Expression

**Figure 7:**
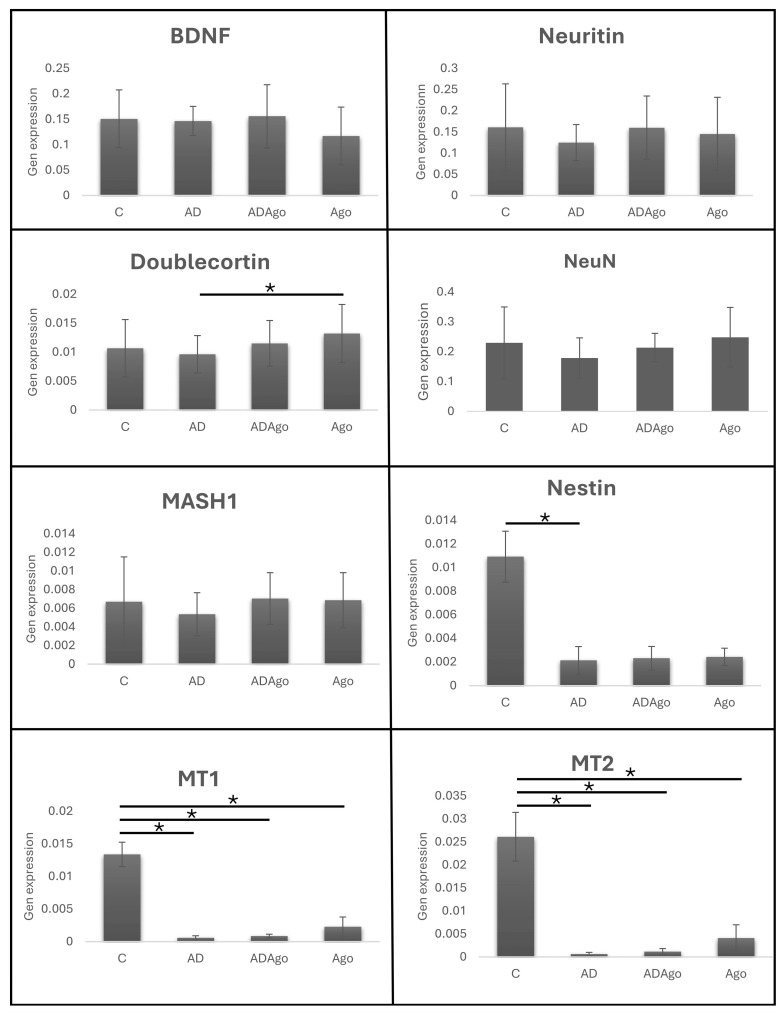
BDNF, Neuritin, DCX, NeuN, MASH1, Nestin, MT1, and MT2 gene analyses were performed in hippocampus tissues using Realtime PCR. No significant difference was observed among the groups in Neuritin and BDNF gene expression levels (*p* > 0.05). A significant difference was observed between AD and Ago groups in DCX gene expression (*p* = 0.049). No significant difference was observed among the groups in NeuN gene expression (*p* > 0.05). No significant difference was observed among the groups in MASH1 gene expression (*p* > 0.05). A significant difference was observed between C and AD in Nestin gene expression (*p* = 0.048). A significant difference was observed between C and AD (*p* = 0.009), C and ADAgo (*p* = 0.01), C and Ago (*p* = 0.028) in MT1 gene expression (*p* < 0.05). A significant difference was observed between C and AD (*p* = 0.023), C and ADAgo (*p* = 0.026), C and Ago (*p* = 0.048) in MT2 gene expression (*p* < 0.05). C: control; AD: Alzheimer’s disease-like; ADAgo: Alzheimer’s disease-like + agomelatine; Ago: agomelatine. * *p* < 0.05.

BDNF, Neuritin, DCX, NeuN, MASH1, Nestin, MT1, and MT2 gene analyses were performed in hippocampus tissues using Realtime PCR. No significant difference was observed among the groups in Neuritin and BDNF gene expression levels (*p* > 0.05). A significant difference was observed between AD and Ago groups in DCX gene expression (*p* = 0.049). No significant difference was observed among the groups in NeuN gene expression (*p* > 0.05). No significant difference was observed among the groups in MASH1 gene expression (*p* > 0.05). A significant difference was observed between C and AD in Nestin gene expression (*p* = 0.048). A significant difference was observed between C and AD (*p* = 0.009), C and ADAgo (*p* = 0.01), and C and Ago (*p* = 0.028) in MT1 gene expression (*p* < 0.05). A significant difference was observed between C and AD (*p* = 0.023), C and ADAgo (*p* = 0.026), and C and Ago (*p* = 0.048) in MT2 gene expression (*p* < 0.05) (Figure 7).

### 3.6. Histological Parameters

**Figure 8:**
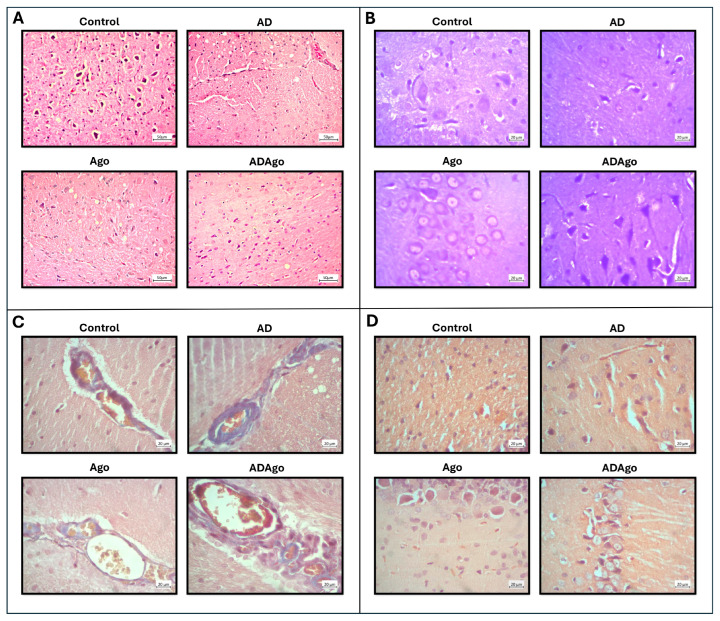
In H&E and Toluidine staining of the AD group there was neuron degeneration and neuron loss, especially a decrease in the Purkinje cell layer. The Ago group had a structure similar to the C group. In the ADAgo group, it was determined that the damage continued but the neuron loss was less than AD group (**A**,**B**). In Masson staining of the AD group, increased collagen fibers were observed in the walls of blood vessels compared to C group. In the Ago group, these collagen fibers were observed similar to the C group. Finally, in the ADAgo group, compared to AD group, a decrease in the amount of collagen fibers in the blood vessel walls was detected (**C**). In Congo Red staining, no apparent amyloid plaque structure was observed in the Purkinje layers of the control and Ago groups. In the AD group, red-stained amyloid plaque structure was detected around the neurons in this layer. In the ADAgo group, the presence of amyloid plaques was noted in this layer, but the number of these plaques was less than in AD group (**D**). C: control; AD: Alzheimer’s disease-like; ADAgo: Alzheimer’s disease-like + agomelatine; Ago: agomelatine.

In the light microscopic examination, brain tissues belonging to the control group were observed as normal in H&E and Toluidine staining. In the brain tissues of the AD group, it was noted that there was neuron degeneration and neuron loss, especially a decrease in the Purkinje cell layer. In addition, congestion and hemorrhage were observed in the vessels in the AD group. The Ago group had a structure similar to C group. In ADAgo group, it was determined that the damage continued but the neuron loss was less than the AD group (Figure 8A,B).

Masson staining is used to stain collagen connective tissue fibers and to show fibrosis. In the C group, blue-stained collagen fibers were observed in the walls of blood vessels. In the AD group, increased collagen fibers were observed in the walls of blood vessels compared to the C group. In the Ago group, these collagen fibers were observed similar to C group. Finally, in ADAgo group, compared to AD group, a decrease in the amount of collagen fiber in the blood vessel walls was detected (Figure 8C).

Congo Red staining is another type of staining used to show amyloid fibrils since it shows protein oligomerization. No apparent amyloid plaque structure was observed in the Purkinje layers of the control and Ago groups. In the AD group, red-stained amyloid plaque structure was detected around the neurons in this layer. Finally, in the ADAgo group, the presence of amyloid plaques was noted in this layer, but the number of these plaques was less than in the AD group (Figure 8D).

## 4. Discussion

This project aimed to investigate effects of Ago on behavior and cognitive functions through neuroprotective mechanisms in an AD-like rat model. For this purpose, the OF test, MWM test, and FST were performed to evaluate behavioral and cognitive functions in AD model rats. Brain tissues were examined histopathologically to demonstrate the damage caused by Aβ. Neuritin, Nestin, DCX, NeuN, BNDF, MASH1, MT1, and MT2 genes were analyzed to evaluate neuroprotective effects and neurogenesis development. In this study, the weights of the experimental animals increased over time. No significant difference was observed among the groups. This may suggest that the rats had similar weights, and their weights would not affect locomotor activity between the groups.

Ago, a melatonin analogue, exerts its antidepressant effects by agonizing MT1 and MT2 receptors while blocking 5-HT_2_C receptors [25].

In addition to improving mood, prolonged Ago treatment has been shown to confer neuroprotection, bolster learning capacity, engage molecular memory–storage pathways, and enhance episodic recall [26,27,28]. In rodent models, Ago facilitates object-recognition memory through its combined melatonergic and serotonergic actions. Clinical data to date report no adverse impact of Ago on memory function in humans [29].

In one investigation, a seven-day course of Ago (40 mg/kg/day) mitigated lipopolysaccharide-induced cognitive and learning deficits in rats [30]. Another study reported distinct anxiolytic and antidepressant effects after both short-term (40 mg/kg for five days) and long-term (40 mg/kg for five weeks) Ago administration, with enduring improvements in motor activity and reduced anxiety persisting two months post-treatment in pinealectomized rats [31]. In models of chronic mild stress, both Ago and melatonin enhanced object recognition and MWM performance, suggesting that Ago not only prevents stress-related hippocampal memory loss but also engages molecular pathways critical for memory consolidation [25].

It has been shown that stress-induced memory impairment can be reversed in radial arm water maze in rats following chronic Ago treatment [26]. Ago modulates glutamate, a major excitatory neurotransmitter involved in neuroplasticity and memory processes. [27]. Ago treatment caused a significant increase in memory and learning performance in diabetic rats [32]. It was investigated whether Ago was effective against behavioral, biochemical, and histological damage in a streptozotocin-induced experimental AD model. Chronic Ago treatment reduced anxiety-like behavior of rats in elevated plus maze test and corrected spatial memory impairment in radial arm maze test. It also corrected biochemical changes such as increased Aβ protein and inflammatory markers (TNF-alpha and IL-1 beta). Ago is thought to be investigated as a therapeutic option as it improves the behavioral symptoms and accompanying neuropathological events observed in the sporadic AD model [33]. Ago has also been found to be beneficial for motor dysfunction in other neurodegenerative diseases such as Parkinson’s disease [34]. Animals treated with Ago showed cognitive improvements in MWM [35].

In the present study, number transitions from edge to center and first transition time from edge to center were examined in OF. No significant changes were observed in the parameters evaluated according to time. This may suggest that locomotor activities similar in the groups. In the evaluation of depression behavior, swimming time, climbing time, immobilization time, and mobility percentage were analyzed in an FST. While high swimming time and climbing time are findings that suggest against depression, high immobilization times are findings that suggest in favor of depression. While depression-like results were observed in the AD group in this study, the opposite was the case in the C, ADAgo, and Ago groups. The highest swimming time was observed in the C group, the highest climbing time was observed in the ADAgo group, the highest immobilization time was observed in the AD group, and the lowest mobility percentage was observed in the AD group. This shows that depressive symptoms occurred in the AD group, and these were improved with the antidepressant effect of Ago. MWM test was performed for learning and memory evaluation in this study. In the training phase, it was observed that the AD group failed to learn the location of platform. In test phase, it was observed that the AD group had memory impairment, and this situation was improved with Ago. Ago showed an improving effect on cognitive functions in Aβ-induced AD model rats. It should be noted that, beyond its agonist action at MT1 and MT2, Ago also acts as an antagonist at serotonin 5-HT_2_C receptors. This serotonergic activity may have contributed to the observed behavioral improvements, particularly in affective domains such as anxiety and depression-like behaviors. Therefore, the behavioral outcomes observed in this study may result from a combination of both melatonergic and serotonergic mechanisms, and the specific contribution of each pathway warrants further investigation.

It has been reported that hippocampal neuron loss is observed histologically after hematoxylin and eosin staining in AD. They reported that neurons in the CA1 region were in an abnormal arrangement compared to the control group, that their cell morphology was abnormal and that they contained many astrocytes and microglia [36]. When Salima Douichene et al. examined the histological results of their study, they found that there was no neuron loss in control group, while in experimental groups, the cytoplasm of neurons collapsed, dark spots were detected in their nuclei, and neurofibrillary degeneration was accompanied by neuron and hippocampus degeneration [37]. Several techniques are available for the detection of Aβ plaques, each with distinct advantages and limitations. Among these, Congo Red staining stands out as a practical and rapid approach for identifying Aβ deposits in vitro. Its affordability also makes it a commonly preferred method in routine histopathological assessments [37]. In a separate study, histological analysis demonstrated that treatment with minocycline (administered at doses of 50 and 100 mg/kg for 30 days) alleviated pathological alterations and significantly reduced Aβ plaque accumulation in the CA1 region of the hippocampus, as evidenced by Congo Red staining [38].

Administration of Ago led to notable increases in both the volume and number of granule and pyramidal neurons within the dentate gyrus and CA1–3 hippocampal subfields, respectively [35]. Ago reduced neuronal loss in the hippocampus subregions of diabetic animals [32]. Aβ1-42 oligomers are known to trigger intense oxidative stress, inflammatory responses, and neuronal cell senescence, all of which contribute to progressive neurodegeneration. Ago has been reported to exert both antioxidant and anti-inflammatory effects. Specifically, it mitigated Aβ1-42-induced neuroinflammation by downregulating proinflammatory cytokines such as TNF-α and IL-1β. Furthermore, Ago counteracted cellular senescence by preventing the reduction in telomerase activity typically induced by Aβ1-42 oligomers [11]. It has been determined that Ago has a neuroprotective function in AD-related pathological damage [10]. Ago reduced hippocampal Aβ accumulation by controlling BACE1 activity [30]. Recent research has demonstrated that prolonged administration of the atypical antidepressant ago in a rat model of AD induced by intracerebroventricular streptozotocin alleviates depressive-like behaviors and spatial memory deficits. These effects are thought to result from the suppression of key AD-related pathologies, including elevated extracellular Aβ levels, increased proinflammatory cytokine expression, and hippocampal neuronal loss [33]. In the present study, histological staining results showed that the morphological structure of neurons in the AD group was impaired and their numbers decreased, consistent with the above studies. It was noted that Ago treatment also improved these damages, albeit slightly.

AD is associated with Aβ accumulation, increased tau hyperphosphorylation, persistent neuroinflammation, and decreased neurotrophic factors, neurogenesis, and synaptic plasticity [39]. Animal studies have shown that Ago treatment increases neuroplasticity mechanisms, neurogenesis, and BDNF levels in hippocampus and prefrontal cortex [40]. Ago activates adult hippocampal neurogenesis by improving hippocampal neuronal activity that is negatively affected by stress [41]. Ago treatment contributes to neurogenesis by inducing upregulation of BDNF mRNA in the prefrontal cortex. This is thought to be due to a synergistic effect between melatonergic and serotonergic receptors and may play a role in memory formation [42]. Melatonin synthesis is irregular in AD. Behavioral abnormalities and hormonal changes occur as a result of neurodegeneration occurring simultaneously before the symptoms of Aβ accumulation due to melatonin synthesis disorder. Melatonin administration has been shown to positively affect the secretion of melatonin in pineal gland and serum levels of growth hormone and IGF-1, BDNF levels in hippocampus, and survival of hippocampal neurons in rats. In addition, melatonin prevented increases in anxiety, locomotor activity, and reference memory damage. Oral administration of melatonin has been considered as a prophylactic strategy to prevent or slow progression of AD pathology [43].

Ago administration increased BDNF levels in rats, improving neuronal survival, neuroplasticity, and neurogenesis [30]. Neuritin plays an important role in neural development and synaptic plasticity. Increased neuritin levels have been shown to attenuate synaptic damage in AD model mice [44,45]. DCX is a microtubule-associated protein used to identify young and immature neurons. DCX is specifically expressed in newly generated healthy neurons, peaking in second week after neonatal birth and decreasing in the fourth week as mature neurons labeled by NeuN emerge [46,47]. Nestin expresses markers indicating neural progenitor cells [48]. In the current study, MT1 and MT2 genes were analyzed for melatonin activity in hippocampus tissues, BDNF and neuritin for synaptic plasticity, and DCX, Neun, Mash1, and Nestin for neurogenesis. Synaptic plasticity and neurogenesis markers were found to be generally low in AD group, although not significant. The low DCX in AD group and the increase in the Ago group represent the increase in young neurons. Although there was no significant difference for NeuN, which indicates mature neurons, it was found to be lower in AD group. The fact that Ago did not create a significant difference in all markers in the neurogenesis mechanism may depend on the stages and the dose of the drug. It is possible that Ago improved cognitive functions not only through neurogenesis but also through its effects on different mechanisms.

The pineal hormone melatonin plays a role in physiologically transmitting temporal information to circadian and seasonal behavioral rhythms and has neuroprotective properties. MT1-MT2, which belong to G protein-coupled receptor family, undergo degeneration in AD, which has serious consequences on neuropathology and clinical symptoms. Immunohistochemical studies have shown a decrease in cellular localization of both melatonin receptors in pineal gland and occipital cortex in AD patients. The decreased melatonin receptor expression in these brain regions suggests that MT1-MT2 may play a role in AD pathology. While both MT1 and MT2 are localized to pinealocytes in the pineal gland, both receptors are expressed in some pyramidal and non-pyramidal cells in the cortex. In patients with AD, parallel to degenerative tissue changes, there is a general decrease in density of receptors in both brain regions and it is thought that it may contribute to AD pathology [49]. It is known that MT receptors affect Aβ neurotoxicity and clearance [50]. Studies have observed a decrease in MT1-MT2 expressions in AD. In the current study, there was a significant difference between C and other groups for MT1-MT2 gene expression. In future studies, it is important to investigate gene expression of these receptors not only in hippocampus but also in different brain regions.

Based on this information, it is thought that Ago shows antidepressant activity in the experimental AD model and may have an effect on cognitive functions through different mechanisms, not only through synaptic plasticity and neurogenesis.

### Limitations

This study has several limitations. Ago was tested at a single dose and treatment duration, limiting insights into dose- or time-dependent effects. The analysis focused solely on the hippocampus, excluding other relevant brain regions such as the prefrontal cortex and amygdala. Additionally, although changes in BDNF and other markers were noted, intra-cellular signaling pathways were not explored in detail. Moreover, this study evaluated only gene expression of biomarkers without assessing protein expression or functional outcomes, which may limit the interpretation of molecular findings. Future studies addressing these points will help clarify the broader therapeutic potential of agomelatine.

## 5. Conclusions

AD is one of the most common neurodegenerative diseases. New options for AD treatment need to be investigated from an economic and social perspective. This study aimed to examine effects of Ago application on neuroprotective mechanisms as well as cognitive and behavioral functions in an experimental AD-like rat model. The results may contribute to research on the pathophysiology and treatment of other neurodegenerative diseases, especially AD. The potential effects of Ago, which is widely used among antidepressant agents, can be considered a promising option for improving cognitive functions in treatment strategies by being investigated in more detail in human and animal models.

## Figures and Tables

**Table 1:** In the force swimming, climbing, immobilization time, and mobility percentage.

Mean ± SD	C	AD	ADAgo	Ago	*p*-Value^2^
Swimming time	162.63 ± 61.01	48.11 ± 32.14	84.00 ± 25.86	141.70 ± 25.2	<0.001
Climbing time	71.50 ± 47.86	96.67 ± 24.89	144.43 ± 34.64	94.80 ± 36.68	0.010
Immobilization time	65.88 ± 31.38	152.22 ± 33.08	71.00 ± 25.87	63.50 ± 32.93	<0.001
Mobility percentage	78.00 ± 10.45	49.23 ± 11.03	76.31 ± 8.61	78.75 ± 10.87	<0.001

**Table 2:** Distance moved, latency to platform, time spent in platform area, velocity, number of passes through target quadrant, time spent in target quadrant, and first transition time to target quadrant parameters were evaluated in the test phase of MWM.

Mean ± SD	C	AD	ADAgo	Ago	*p*-Value^2^
Distance moved (cm)	1006.52 ± 405.43	2413.45 ± 1183.26	851.74 ± 370.05	869.22 ± 365.58	<0.001
Latency to the platform (s)	13.01 ± 8.82	30.19 ± 9.30	16.51 ± 10.41	11.25 ± 6.22	0.019
Velocity	29.63 ± 12.31	35.16 ± 8.55	25.10 ± 11.33	25.24 ± 10.63	0.34
Time spent in the target quadrant (s)	17.19 ± 6.43	11.11 ± 3.01	16.94 ± 5.99	17.48 ± 5.37	0.039
First transition time to target quadrant (s)	6.27 ± 4.59	4.57 ± 2.59	7.45 ± 3.86	2.18 ± 1.37	0.024

## Data Availability

The data are available from the corresponding author upon reasonable request.

## Supplementary Materials

The following supporting information can be downloaded at: https://www.mdpi.com/article/10.3390/medicina61081315/s1, Figure S1. Melting curve analysis of primers used for qPCR; Table S1. List of primers used for qPCR [21,22,23].
