# Supplementary material for: Agomelatine Ameliorates Cognitive and Behavioral Deficits in Aβ-Induced Alzheimer’s Disease-like Rat Model
Source: Medicina (Kaunas). 2025 Jul 22;61(8):1315. doi: 10.3390/medicina61081315 (PMC12388420; doi:10.3390/medicina61081315)

Table S1. List of primers used for qPCR

Gene	Primer sequence (5’->3’)	Amplicon size (bp)	Reference
**Neuritin**	TCGCGGTGCAAATAGCTTAC<br>CGGTCTTGATGTTCGTCTTGTC	152	[21]
**Nestin**	CACACCTCAAGATGTCCCTTAG<br>AGGTACTGGTCCTCTGGTATC	166	[21]
**DCX**	AGGTACGTTTCTACCGCAATG<br>CTGAGGCAGGTTGATGTTGT	137	[21]
**NeuN**	GGCAAATGTTCGGGCAATTC<br>GATCGTCCCATTCAGCTTCTC	140	[21]
**BDNF**	CTGAGCGTGTGTGACAGTATTA<br>GGGATTACACTTGGTCTCGTAG	153	[21]
**MASH1**	GTTCGGCGGTCGAATACAT<br>GAGTTCAAGTCGTTGGAGTAGTT	127	[21]
**MT1**	GGCGCTGACGTCTATACTTAAC<br>CGATCCCGGTGATGTTGAATAC	116	This study
**MT2**	CTCATCCTTGTGGCCATTCT<br>CAATGGCTGTGATGTTGAAGAC	118	This study
**SDHA**	AGCAAGCTCTATGGAGACCT<br>TAATCGTACTCATCAATCCG	200	[22]
**RPL13A**	GGATCCCTCCACCCTATGACA<br>CTGGTACTTCCACCCGACCTC	132	[23]

Figure S1. Melting curve analysis of primers used for qPCR

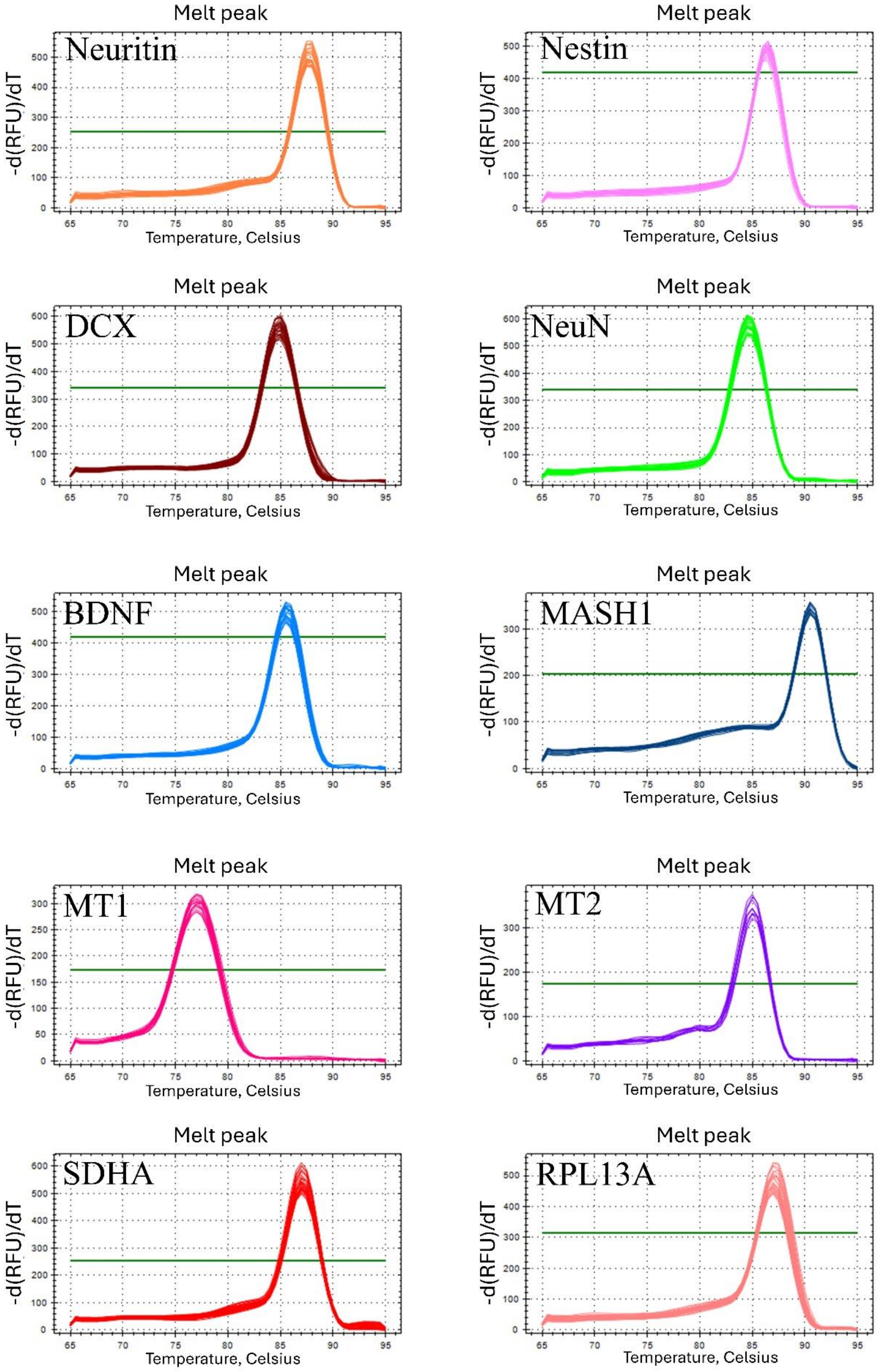

Supplement: Supplementary file 1 [file medicina-61-01315-s001.zip › medicina-3730220-supplementary.pdf]
